# Sestrin 2 attenuates sepsis‐associated encephalopathy through the promotion of autophagy in hippocampal neurons

**DOI:** 10.1111/jcmm.15313

**Published:** 2020-05-04

**Authors:** Lili Luo, Jinlin Wu, Lina Qiao, Guoyan Lu, Jinhui Li, Deyuan Li

**Affiliations:** ^1^ Department of Pediatrics West China Second University Hospital Sichuan University Chengdu China; ^2^ Key Laboratory of Birth Defects and Related Disease of Women and Children Ministry of Education Sichuan University Chengdu China

**Keywords:** autophagy, sepsis‐associated encephalopathy, SESN2, unc‐51‐like kinase 1

## Abstract

Sepsis‐associated encephalopathy (SAE) has typically been associated with a poor prognosis. Although sestrin 2 (SESN2) plays a crucial role in metabolic regulation and the stress response, its expression and functional roles in SAE are still unclear. In the present study, SAE was established in mice through caecal ligation and puncture (CLP). The adeno‐associated virus 2 (AAV2)‐mediated SESN2 expression (*ie* overexpression and knockdown) system was injected into the hippocampi of mice with SAE, and subsequently followed by electron microscopic analysis, the Morris water maze task and pathological examination. Our results demonstrated an increase of SESN2 in the hippocampal neurons of mice with SAE, 2‐16 hours following CLP. AAV2‐mediated ectopic expression of SESN2 attenuated brain damage and loss of learning and memory functions in mice with SAE, and these effects were associated with lower pro‐inflammatory cytokines in the hippocampus. Mechanistically, SESN2 promoted unc‐51‐like kinase 1 (ULK1)‐dependent autophagy in hippocampal neurons through the activation of the AMPK/mTOR signalling pathway. Finally, AMPK inhibition by SBI‐0206965 blocked SESN2‐mediated attenuation of SAE in mice. In conclusion, our findings demonstrated that SESN2 might be a novel pharmacological intervention strategy for SAE treatment through promotion of ULK1‐dependent autophagy in hippocampal neurons.

## 1 INTRODUCTION

Sepsis is caused by a non‐homeostatic response of the host to an infection and is a major clinical challenge associated with multi‐organ dysfunction.^1^ Previous clinical studies reported that the brain is one of the first organs to be affected by sepsis, and up to 60% of sepsis survivors exhibit permanent cognitive deficits and memory loss. This phenomenon is referred to as sepsis‐associated encephalopathy (SAE).^2^ Specifically, SAE is the most common form of encephalopathy occurring in critical care settings and has often been related to poor prognosis.^3^, ^4^, ^5^

Autophagy is an evolutionarily conserved catabolic recycling process, involving degradation of either damaged or senescent organelles and proteins.^6^ Autophagy is induced in sepsis by the binding of pathogen‐associated molecular pattern molecules to pattern recognition receptors within a microbial structure.^7^ In fact, a considerably increased number of autophagosomes, as well as higher LC3‐II/LC3‐I ratios, were observed in hepatocytes^8^ and cardiomyocytes^9^ of mice with sepsis. Additionally, the deletion of ATG genes enhanced the production of pro‐inflammatory cytokines, including interleukin 1β (IL‐1β), in multiple organ tissues following CLP.^10^ Similarly, autophagosome formation and lysosome activation increased in the hippocampi of Wistar rats after CLP. These changes were associated with an increase of LC3II, and a reduction in Beclin‐1, LAMP1 and RAB7.^11^

SESN2 belongs to the evolutionarily conserved sestrin family, which is involved in metabolic regulation and stress response.^12^, ^13^ Previous studies indicated that SESN2 was transcriptionally regulated by p53^14^ and activated by hypoxia in neonatal rats.^15^ Moreover, accumulating evidence suggested that SESN2 played a crucial role in regulating cell proliferation, apoptosis and autophagy in various cancers.^16^, ^17^, ^18^ Finally, genetic ablation of SESN2 enhanced hair cell sensitivity to gentamicin, confirming the protective role of SESN2 in gentamicin‐induced stress.^19^ Notably, despite the significant progress made in understanding the implications of SESN2 in metabolic pathways and diseases, its functional role and underlying mechanisms in SAE are yet to be investigated.

Caecal ligation and puncture, that is (CLP)‐induced polymicrobial sepsis, is the most frequently used sepsis model due to a high degree of similarity to human sepsis characteristics.^20^ In the present study, the SAE model was established through CLP in C57 mice, and in vivo and in vitro experiments were performed to explore the expression and function of SESN2 during SAE development. Results demonstrated that SESN2 protected against SAE by induction of ULK1‐dependent autophagy with subsequent attenuation of inflammation in hippocampal neurons of mice with SAE.

## 2 MATERIALS AND METHODS

### 2.1 Sepsis‐associated encephalopathy (SAE) model and treatment

C57 mice (female, 6‐8 weeks old, 8 mice per group) were purchased from the Hfkbio Company (Beijing, China) and acclimated to a 12‐h day/night cycle, under specific pathogen‐free and ad libitum food intake conditions, at least 1 week prior to initiating the experiments. Subsequently, SAE was induced by caecal ligation and puncture (CLP), as previously described.^21^ Briefly, mice were first anaesthetized through an intraperitoneal injection of 4% diethyl ether. Subsequently, the caecum was ligated distal to the ileocecal valve without any intestinal obstruction and punctured with a 20‐gauge needle at two sites, followed by the expression of a small amount of faecal material into the peritoneal cavity. In contrast, caecal ligation and puncture were not performed on sham mice, although the same procedure was conducted. Subsequently, mice were killed at indicated time‐points following SAE development, and hippocampal tissues were collected for analysis. Finally, the AAV2‐Ctrl (empty vector control), AAV2‐SESN2, AAV2‐shSESN2 (1 × 10^10^ GU in 1 μL) and SBI‐0206965 (specific AMPK inhibitor, 2 mg/kg, total volume 1 μL) were intracranially injected 2 hours prior to the CLP operation with a 30‐gauge needle. The Institutional Animal Care and Use Committees of the Sichuan University approved all experimental procedures.

### 2.2 Electron microscopic analysis

Electron microscopic analysis was performed as previously indicated.^22^ Specifically, 8 hours after the CLP operation, the brains of mice with SAE were perfused and fixed with 4% paraformaldehyde and 1% glutaraldehyde for 48 hours. Following fixation, they were cut into thin sections (40‐60 μm) and mounted onto copper grids. The sections were contrasted with uranyl acetate and Reynold's lead citrate, and then examined with the Philips CM 10 electron microscope. Finally, autophagosomes were detected in six random sections and analysed.

### 2.3 Western blotting

Isolated mouse brain tissues were collected at 0, 2, 4, 8, 12 and 16 hours after CLP surgery and homogenized in an ice‐cold lysis buffer (Beyotime, Beijing, China) containing several protease inhibitors (Merck Millipore, MA, USA). Thereafter, the lysates were centrifuged at 12 000 g for 15 minutes at 4°C, and the protein concentrations were determined with a Bicinchoninic Acid Protein Assay Kit (Beyotime, Beijing, China). The protein samples (10 µg/lane) were then separated on 8%‐12% sodium dodecyl sulphate‐polyacrylamide (SDS‐PAGE) gels and electroblotted onto polyvinylidene fluoride (PVDF) membranes. Following blotting, the PVDF membranes were blocked with 5% non‐fat dry milk in a TBS/T buffer and incubated overnight at 4°C with primary antibodies against the following proteins: SESN2 (PA5‐72834; Thermo Fisher, MA, USA), p‐AMPK (ab32047; Abcam, CA, UK), AMPK (5831; CST Tec., MA, USA), p‐mTOR (ab109268; Abcam, CA, UK), mTOR (2972; CST Tec.), p‐ULK1 (ab203207, Abcam, CA, UK), ULK1 (8054) and GAPDH. Subsequently, the PVDF membranes were incubated with either peroxidase‐conjugated goat anti‐rabbit or antimouse immunoglobulin G (IgG) antibodies (ZSBIO, Beijing, China), at 37°C for 1 hours. Finally, enhanced chemiluminescence (Merck Millipore, MA, USA) was used to detect bound antibody signals with the iBright Imaging System (Thermo Fisher, MA, USA), and the visualized protein band densities were quantified with Image‐Pro Plus imaging software (version 6.0; Media Cybernetics, Inc, Rockville, MD, USA).

### 2.4 Haematoxylin and eosin, and immunofluorescence staining

Sections of the formalin‐fixed paraffin‐embedded brain tissue specimens were cut into 4 μm slides, dewaxed with xylene (twice, 15 minutes each time) and hydrated in an alcohol gradient (100%, 95%, 85% and 75%, 5 minutes in each concentration). Thereafter, the slides were stained with haematoxylin and eosin (H&E) to assess the degree of brain injury. The hydrated slides were processed for antigen retrieval under high temperature and pressure (3 minutes), and blocked with goat serum before immunofluorescence staining. Subsequently, the slides were incubated overnight at 4°C with primary antibodies against the following proteins: SESN2 (PA5‐72834, Thermo Fisher, MA, USA), NeuN (MAB377, EMD Millipore, Billerica, MA, USA), GFAP (MAB360, EMD Millipore Billerica, MA, USA), p‐AMPK (ab32047, Abcam, CA, UK), p‐mTOR (ab109268, Abcam, CA, UK) and p‐ULK1 (ab203207, Abcam, CA, UK). The sections were then incubated with either fluorescein isothiocyanate/tetramethylrhodamine‐conjugated antimouse, or rabbit IgG antibodies (Thermo Fisher, MA, USA), at 37°C for 1 hour. Finally, nuclei were stained with 4',6‐diamidino‐2‐phenylindole (DAPI) (Beyotime, Beijing, China), and a total of 6 sections per group were analysed under a DX51 microscope (Nikon Corporation, Tokyo, Japan).

### 2.5 TUNEL assay

A TUNEL assay to detect apoptotic cells in the brain tissues was conducted with a DeadEnd Fluorometric TUNEL System (Promega Corporation, Madison, WI, USA) according to the manufacturer's protocol. Dark‐green fluorescent staining visualized with fluorescence microscopy identified the TUNEL‐positive nuclei stained with DAPI (Beyotime Bio, China, 1:100). We then calculated the percentage of TUNEL‐positive cells in each section, and six random frames were analysed per group.

### 2.6 Morris water maze (MWM) task

The MWM task was performed as previously indicated.^23^ Briefly, mice were trained for the MWM task from the 3rd to 6th day (four times per day) following CLP, in a round white pool containing a 25‐cm^2^ hidden escape platform (1 cm below the water level). Each trial began by placing the mice on a platform for 20 seconds, to allow orientation to the extra maze cues. Next, the mice were gently lowered (tail‐first) into the pool facing the wall at one of three positions, and the elapsed time while swimming to the platform was recorded. The spatial reference memories of the mice were evaluated 24 hours after the last training session with a probe test, and the platform removed from the pool. All mice were monitored for 60 seconds to observe the swimming distance, time spent in the target quadrant and frequency of crossing the platform.

### 2.7 Detection of cytokine expression

The isolated mouse brain tissues were homogenized in an ice‐cold lysis buffer (Beyotime, Beijing, China), containing several protease inhibitors (Merck Millipore, MA, USA). Next, the lysates were centrifuged at 12 000 g for 15 minutes at 4°C, and the protein concentrations were determined with a Bicinchoninic Acid Protein Assay Kit (Beyotime, Beijing, China). The protein samples (100 µg/lane) were dispensed into ELISA plate wells for measurement of IL‐6, TNF‐α, IL‐10 and IL‐1 β cytokine expression, according to the manufacturer's instructions (eBioscience, MA, USA).

### 2.8 Statistical analysis

All data were expressed as mean ± SD. One‐way ANOVA and independent *t* tests were conducted to compare the differences between the groups, and Dunnett's test was performed for multiple comparisons correction. A two‐way ANOVA with Bonferroni correction analysed latency, distance and time during the water maze training. A value of *P* < 0.05 was considered statistically significant.

## 3 RESULTS

### 3.1 Increase in SESN2 expression in hippocampal neurons following SAE

**Figure 1:**
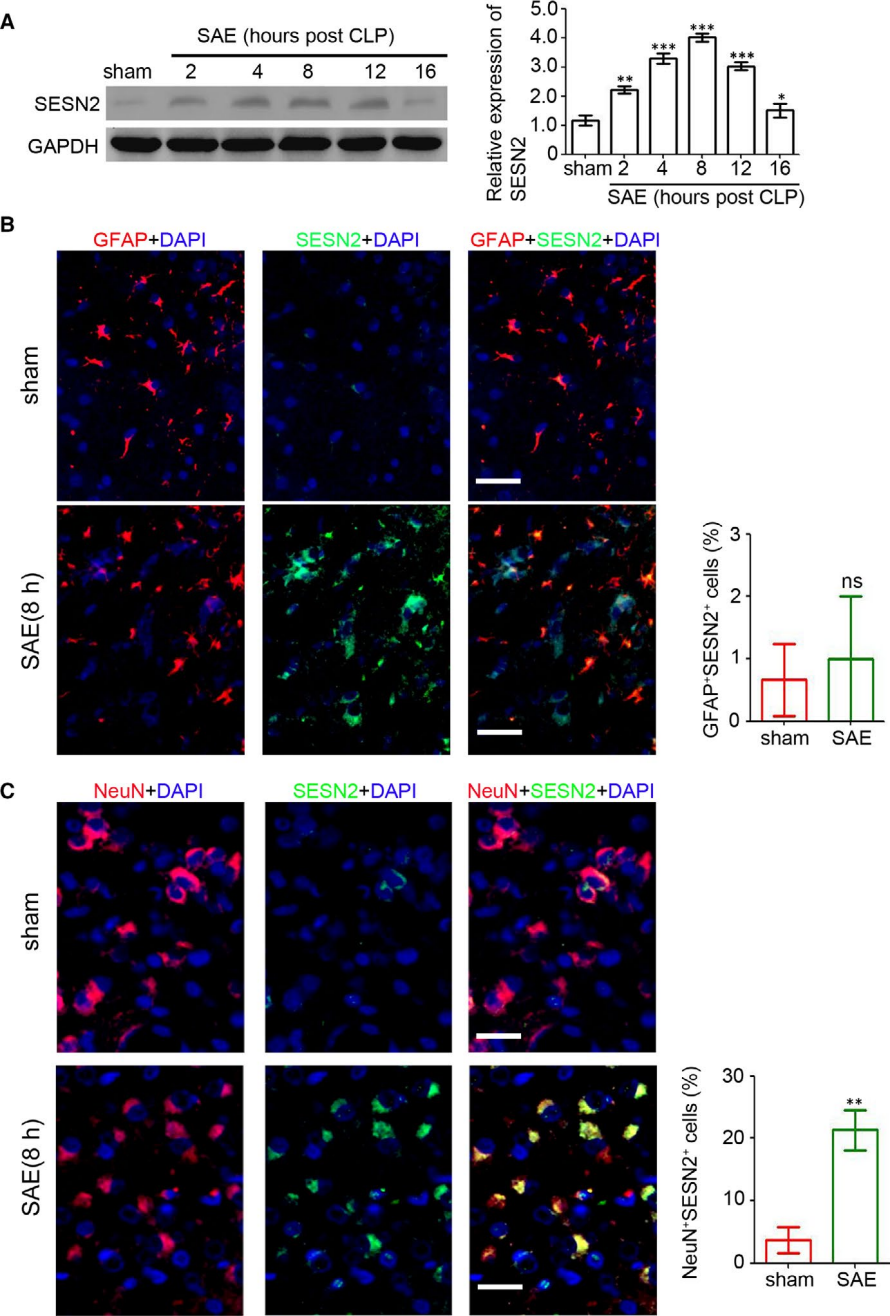
Up‐regulation of SESN2 in neurons following SAE. The SAE model was established in C57 mice by ligation and puncture (CLP). The brain tissues were collected at 0, 2, 4, 8, 12 and 16 hours following CLP. A, SESN2 expression in the brain tissues was determined by Western blotting. GAPDH was used as loading control. The relative expression of SESN2 was analysed (n = 4, **P < 0.01; ***P < 0.001, compared with the sham group; independent *t* test). Immunofluorescence staining of SESN2 (green) and either GFAP (red, B) or NeuN (red, C) in the brain tissues of C57 mice with CLP. The percentage of double‐positive cells was assessed. Scale bar = 100 μm. (n = 6, ns, no significant difference; **P < 0.01, compared with the sham group; independent t test)

The SAE model was established in C57 mice *via* CLP to investigate the expression of SESN2 during SAE development. Mouse brain tissues were collected at 0, 2, 4, 8, 12 and 16 hours following the surgery. Our results suggested a significant increase in SESN2 2‐16 hours after CLP, peaking at 8 hours (Figure 1A). We further explored expression and distribution of SESN2 in brain tissues after SAE development with a double immunofluorescence stain to detect SESN2 protein and the astrocyte‐specific marker GFAP, or the neuron‐specific marker NeuN. Results demonstrated that SESN2‐positive cells colocalized with neurons (Figure 1C), and only a small amount of SESN2 was observed in astrocytes (Figure 1B). Overall, the current study revealed a dramatic up‐regulation of SESN2 expression in the neurons of mice with SAE.

### 3.2 Overexpression of SESN2 attenuates SAE‐related damage

**Figure 2:**
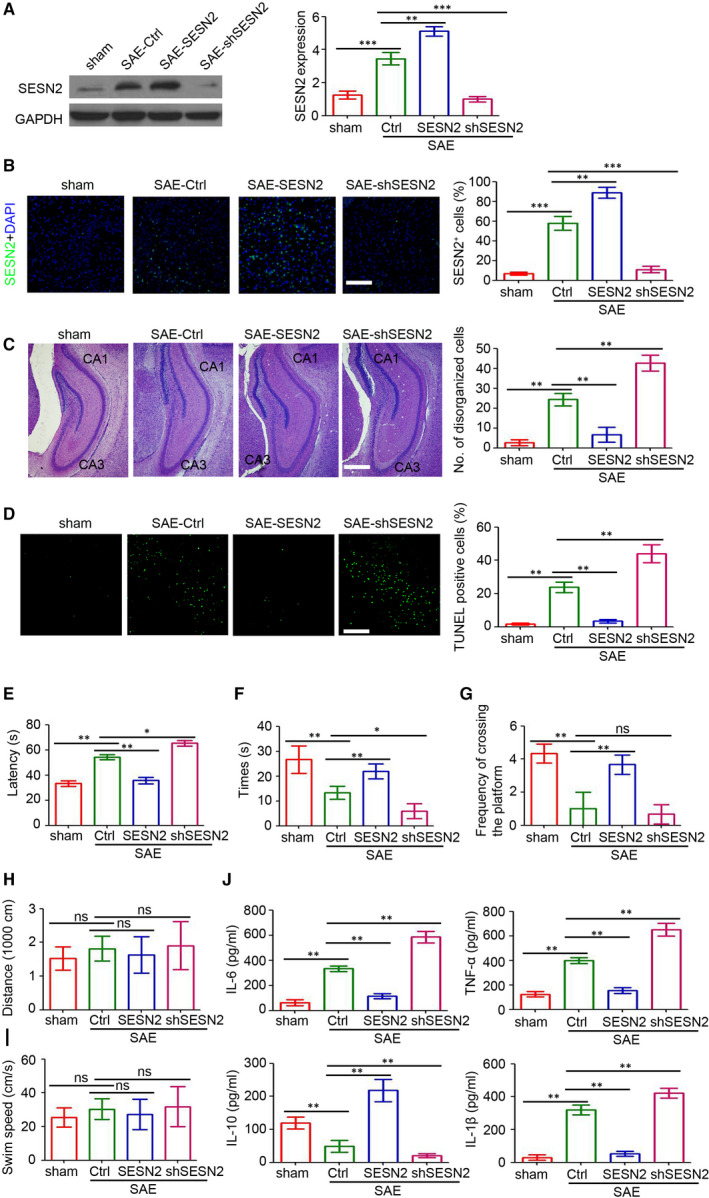
Overexpression of SESN2 inhibits SAE‐related damage. The AAV2‐Ctrl, AAV2‐SESN2 and AAV2‐shSESN2 were separately injected into the hippocampus of C57 mice at two hours prior to the CLP operation. A, SESN2 expression in the brain tissues was determined by Western blotting. GAPDH was used as loading control. The relative expression of SESN2 was analysed (n = 4, **P < 0.01). B, Immunofluorescence staining of SESN2 (green) in the brain tissues. The percentage of SESN2‐positive cells was assessed. Scale bar = 200 μm (n = 6, **P < 0.01; ***P < 0.001. C, Light microscopy of the brain tissues in different groups (haematoxylin and eosin (H&E), scale bar = 500 μm). The number of disorganized cells in whole hippocampus (CA1 and CA3) was counted and evaluated (n = 6, **P < 0.01). D, Detection of apoptotic cells in the hippocampus of mice by TUNEL assay. The per cent of TUNEL‐positive cells were assessed. Scale bar = 200 μm (n = 6, **P < 0.01). E, The time required by mice to reach the platform was measured in the Morris water maze task (n = 4, **P < 0.01). F, The time spent in the target quadrant was measured to assess the memory retention capabilities in Morris water maze task (n = 4, *P < 0.05; **P < 0.01). G, The frequency of mice crossing the platform area was recorded (n = 4, ns, no significant difference;**P < 0.01). H, The distance travelled of mice in 60 s was recorded (n = 4, ns, no significant difference). I, The swim speed of mice in 60 s was recorded (n = 4, ns, no significant difference). J, Cytokine (IL‐6, TNF‐α, IL‐10, IL‐1β) expression in the brain tissues of SAE mice (n = 4, **P < 0.01)

We further explored the functional role of SESN2 during SAE development by injecting the AAV2‐mediated SESN2 overexpression and knockdown systems into mouse hippocampi 2 hours prior to the CLP procedure. Eight hours post‐CLP surgery, results showed that AAV2‐SESN2 increased SESN2 expression in the brain tissues of mice with SAE (Figure 2A), whereas AAV2‐shSESN2 dramatically inhibited it. Furthermore, the results were confirmed by immunofluorescence staining (Figure 2B). Additionally, pathological examinations showed that increased neuronal damage, pericellular/perivascular oedema and inflammatory cell infiltration were found in AAV2‐shSESN2‐injected mice, whereas ectopic SESN2 expression attenuated SAE‐induced brain damage (Figure 2C). The TUNEL assay results also revealed fewer damaged cells in AAV‐SESN2‐injected mice and a greater number of damaged cells in AAV‐shSESN2‐injected mice (Figure 2D). In other experiments, we performed the Morris water maze task to investigate learning and memory functions in mice with SAE. As shown in Figure 2E, mice that underwent the CLP surgery required more time to find the platform than sham group mice, 6 days post‐surgery. Notably, mice in the AAV2‐SESN2 group required less time to find the platform than mice in the SAE‐Ctrl group, whereas mice in the AAV2‐shSESN2 group (Figure 2E) required more time. After the water maze training, the platform was removed, and the swimming distance, time spent in the target quadrant and the platform‐crossing frequency were recorded. Our results showed that mice in the AAV2‐SESN2 group also remained longer in the target quadrant where the platform was located, and crossed the platform more frequently, whereas the opposite was true for AAV2‐shSESN2‐injected mice with SAE (Figure 2F,G). However, the distance travelled and swim velocity were similar in the four groups (Figure 2H,I). Finally, cytokine detection confirmed lower inflammation in mice with SESN2 overexpression *versus* higher inflammation in AAV2‐shSESN2‐injected mice (Figure 2J). Overall, our results indicated ectopic expression of SESN2 attenuated SAE‐related damage.

### 3.3 SESN2 induces ULK1‐dependent neuronal autophagy in mice with SAE

**Figure 3:**
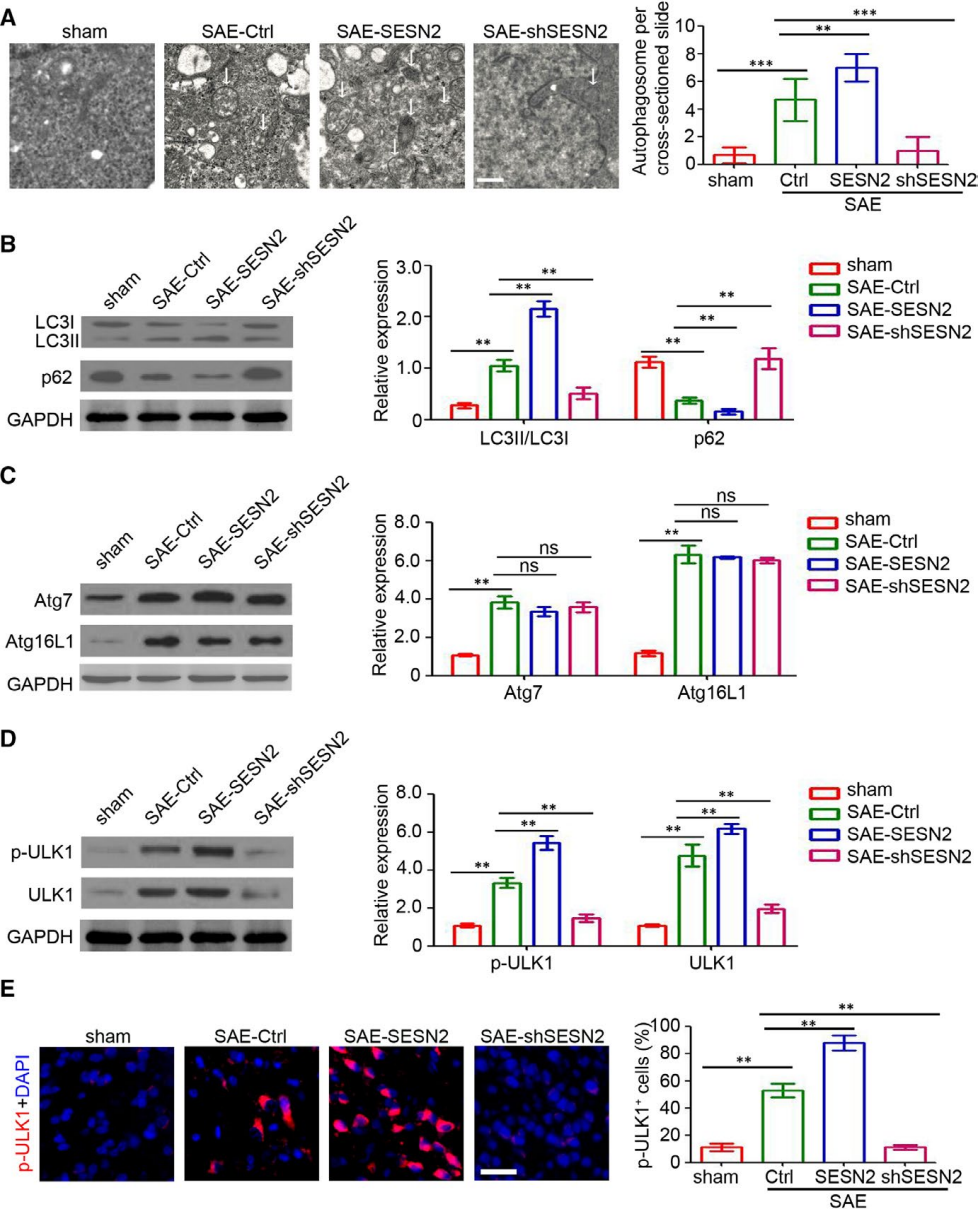
SESN2 promotes the ULK1‐dependent autophagy of neurons. A, Electron microscopy was performed to detect autophagosomes in mouse hippocampi. The white arrow indicates autophagosomes. The number of autophagosomes was assessed (n = 6, **P < 0.01; ***P < 0.01). B‐D, Western blot analysis of the LC3, p62, Atg7, Atg16L1, p‐ULK1 and ULK1 expression in the brain tissues of mice. GAPDH was used as a loading control. The relative expression was analysed (n = 3, ns, no significant difference; **P < 0.01). E, Immunofluorescence staining of the p‐ULK1 (red) in the brain tissues. The percentage of p‐ULK1‐positive cells was assessed. Scale bar = 100 μm. (n = 6, **P < 0.01)

We investigated the potential mechanism behind SESN2 regulation of SAE development by detecting autophagosomes with electron microscopy in the hippocampi of mice with SAE. Our results showed an increased number of autophagosomes in the mice with SAE and an absence of autophagosomes in the sham mice group (Figure 3A). Furthermore, SESN2 overexpression promoted autophagy in the hippocampi of mice with SAE, whereas its knockdown inhibited the formation of autophagosomes (Figure 3A). Additionally, the Western blotting results indicated that SESN2 enhanced LC3II/LC3I expression and inhibited p62 expression (Figure 3B). In contrast, SESN2 did not have significant effects on either Atg7 or Atg16L1 expression in the brain tissues of mice with SAE (Figure 3C). Moreover, an increase in p‐ULK1 and ULK1 expression was found in AAV2‐SESN2‐injected mice, whereas SESN2‐knockdown mice showed decreased expression (Figure 3D). Similarly, immunofluorescence staining confirmed the regulatory role of SESN2 on p‐ULK1 expression in the hippocampi of mice with SAE (Figure 3E). Overall, these findings suggest that SESN2 promotes ULK1‐dependent autophagy in the hippocampi of mice with SAE and thus may be the mechanism underlying SESN2‐induced attenuation of SAE.

### 3.4 SESN2 regulates the AMPK/mTOR signalling pathway in the hippocampi of mice with SAE

**Figure 4:**
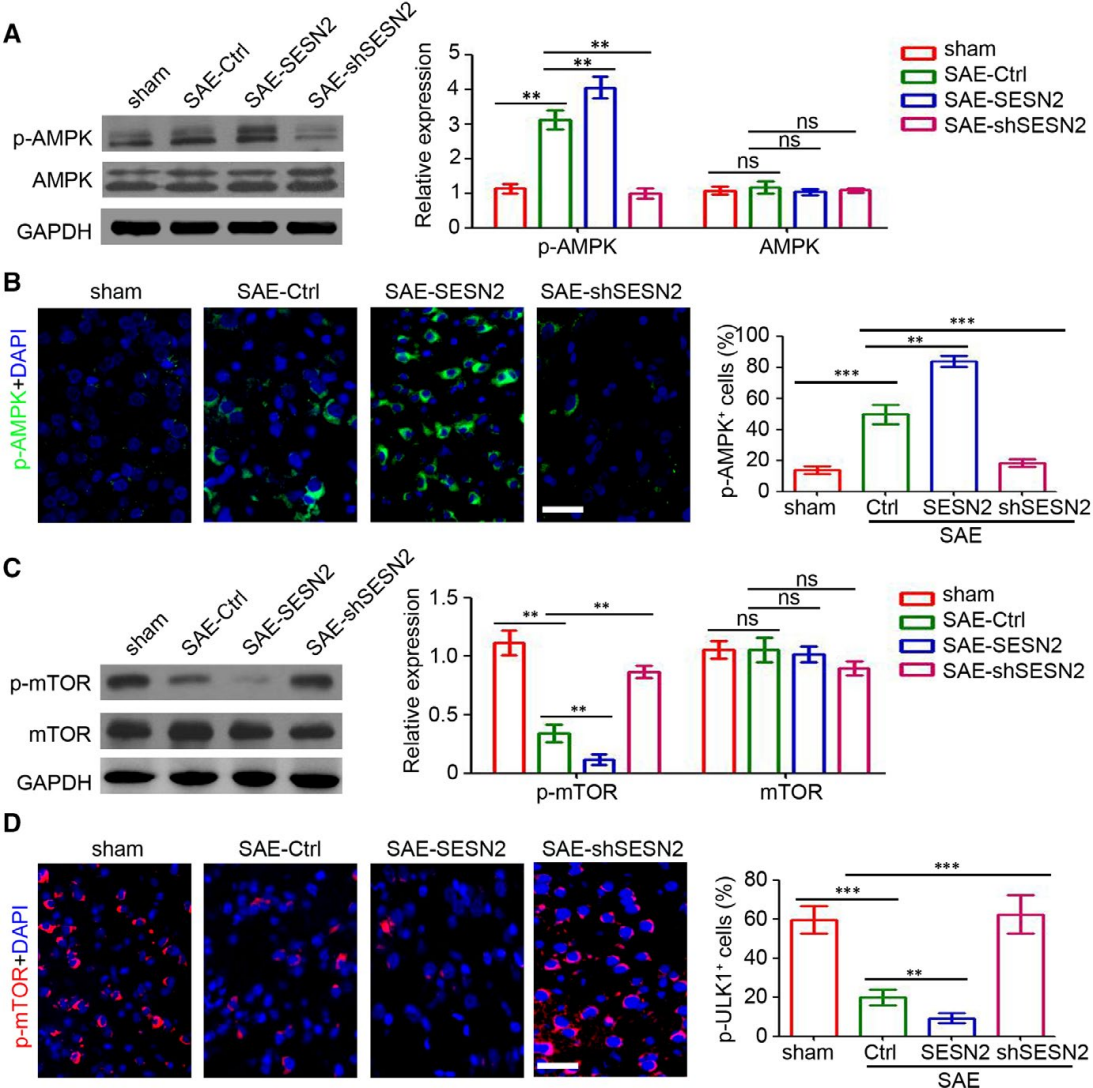
SESN2 regulates the AMPK/mTOR signalling pathway. A, Western blot analysis of both the p‐AMPK and AMPK expression in the brain tissues of mice. GAPDH was used as a loading control. The relative expression was analysed (n = 3, ns, no significant difference; **P < 0.01). B, Immunofluorescence staining of the p‐AMPK (green) in the brain tissues. The percentage of p‐ULK1‐positive cells was assessed. Scale bar = 100 μm. (n = 6, **P < 0.01). C, Western blot analysis of both the p‐mTOR and mTOR expression in the brain tissues of mice. GAPDH was used as a loading control. The relative expression was analysed (n = 3, ns, no significant difference; **P < 0.01). D, Immunofluorescence staining of the p‐mTOR (red) in the brain tissues. The percentage of p‐ULK1‐positive cells was assessed. Scale bar = 100 μm (n = 6, **P < 0.01)

As AMPK is an effector of SESN2, we investigated its expression in mouse hippocampi. As shown in Figure 4A, SESN2 efficiently promoted the activation of AMPK in mouse hippocampi, whereas its knockdown inhibited p‐AMPK expression in mouse brain tissues. Additionally, immunofluorescence staining confirmed the regulatory role of SESN2 on p‐AMPK expression in mouse hippocampi (Figure 4B). As a consequence of AMPK activation, p‐mTOR expression was also regulated by SESN2 (Figure 4C). In fact, fewer p‐mTOR‐positive cells were seen in AAV2‐SESN2‐injected mouse hippocampi than in the shSESN2 group (Figure 4D). Overall, the results showed that SESN2 regulated the AMPK/mTOR signalling pathway in the hippocampi of mice with SAE.

### 3.5 Inhibition of AMPK activation prevents SESN2‐induced autophagy in the hippocampi of mice with SAE

**Figure 5:**
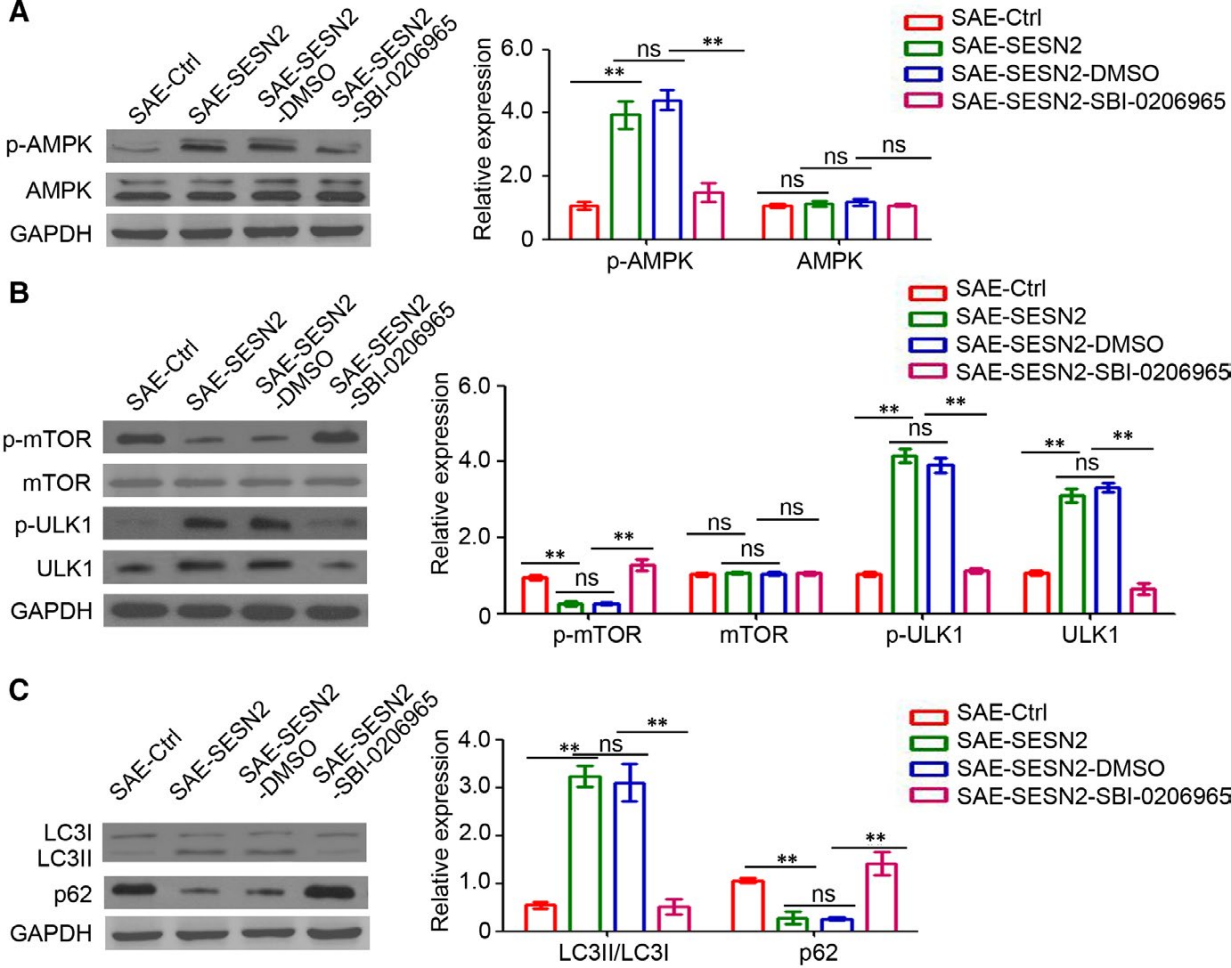
Inhibition of AMPK activation prevents SESN2‐induced autophagy. A, Western blot analysis of the p‐AMPK and AMPK expression in the brain tissues of mice. The relative expression was analysed. B, Western blot analysis of both the p‐mTOR, mTOR, p‐ULK1 and ULK1 expression in the brain tissues of mice. The relative expression was analysed. C, Western blot analysis of both the LC3 and p62 expression in the brain tissues of mice. The relative expression was analysed. GAPDH was used as a loading control (n = 3, ns, no significant difference; ns, no significant difference; **P < 0.01)

We confirmed the role of the AMPK/mTOR signalling pathway in SESN2 regulation of SAE development by treating mice with SBI‐0206965, a specific AMPK activation inhibitor. As illustrated in Figure 5A, SBI‐0206965 efficiently blocked the SESN2‐mediated up‐regulation of p‐AMPK in the hippocampi of mice with SAE. Furthermore, SESN2‐mediated abnormal expression of p‐mTOR, p‐ULK1 and ULK1 was also attenuated after injection of SBI‐0206965 into the hippocampi of mice with SAE (Figure 5B). Given the decrease in p‐ULK1, SESN2‐mediated autophagy in the hippocampi of mice with SAE was also prevented by SBI‐0206965 (Figure 5C). Overall, our results demonstrated that the AMPK/mTOR signalling pathway played a crucial role during SESN2‐induced autophagy in the hippocampi of mice with SAE.

### 3.6 Inactivation of AMPK prevents SESN2‐mediated protection from SAE

**Figure 6:**
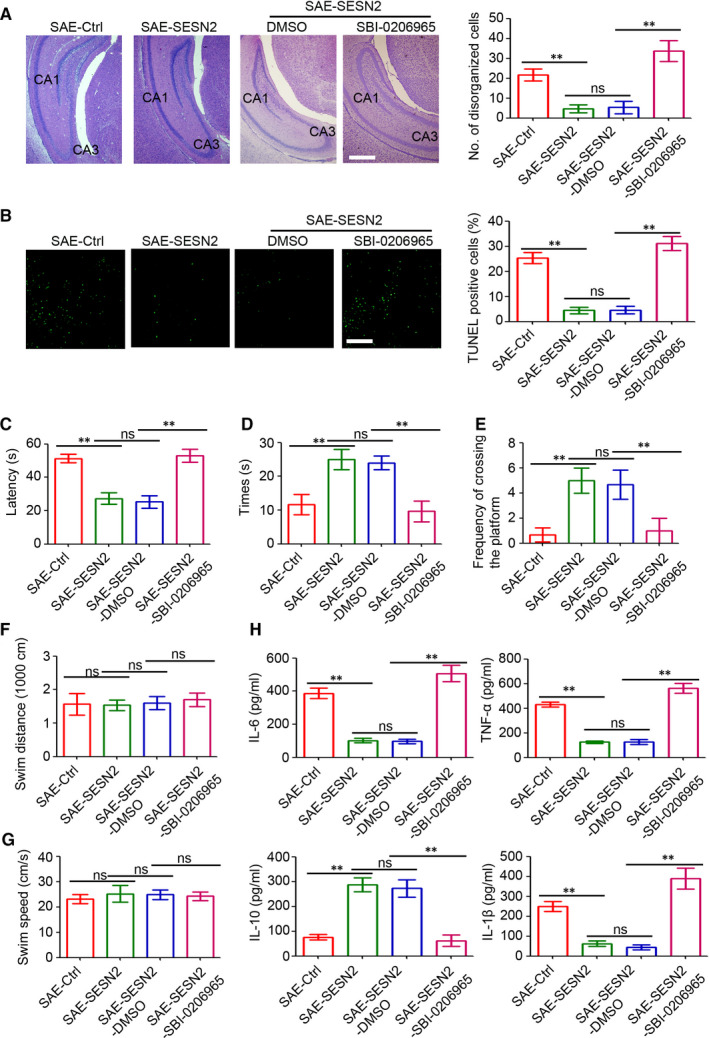
Inactivation of AMPK prevents the SESN2‐mediated protection from SAE. A, Light microscopy of the brain tissues in different groups (haematoxylin and eosin (H&E), scale bar = 500 μm). The number of disorganized cells in whole hippocampus (CA1 and CA3) was counted and analysed (n = 6, ns, no significant difference; **P < 0.01). B, Detection of apoptotic cells in the hippocampus of mice by TUNEL assay. The per cent of TUNEL‐positive cells were assessed. Scale bar = 200 μm (n = 6, **P < 0.01). C, The time required by mice to reach the platform was measured in spatial acquisition trials (n = 4, ns, no significant difference; **P < 0.01). D, The time spent in the target quadrant was measured to assess the memory retention capabilities in the probe trial (n = 4, ns, no significant difference; **P < 0.01). E, The frequency of mice crossing the platform area was recorded (n = 4, ns, no significant difference;**P < 0.01). F, The distance travelled of mice in 60 s was recorded (n = 4, ns, no significant difference). G, The swim speed of mice in 60 s was recorded (n = 4, ns, no significant difference). H, Cytokine (IL‐6, TNF‐α, IL‐10, IL‐1β) expression in the brain tissues of SAE mice (n = 4, ns, no significant difference; **P < 0.01)

As SBI‐0206965 played a role in the inhibition of SESN2‐mediated autophagy, we also evaluated any change in the pathology of mice with SAE. As shown in Figure 6A,B, inhibition of the AMPK/mTOR signalling pathway blocked SESN2‐mediated attenuation of brain damage and apoptosis in mice with SAE. In concordance, the Morris water maze experiments also indicated SBI‐020696‐ inhibition of decreased learning and memory functions in AAV2‐SESN2‐injected mice with SAE (Figure 6C,E), but had no significant effect on the distance travelled or swimming velocity (Figure 6F,G). Additionally, an increase in pro‐inflammatory cytokines was detected in SBI‐0206965‐injected hippocampi of mice with SAE, compared to mice with SESN2 overexpression (Figure 6H). Overall, our findings indicated that the AMPK/mTOR signalling pathway played a crucial role during SESN2‐mediated attenuation of SAE‐induced brain damage.

## 4 DISCUSSION

In the present study, the following results were highlighted: (a) SESN2 was increased in the hippocampal neurons of mice with SAE; (b) ectopic expression of SESN2 by AAV2 efficiently attenuated brain damage and loss of learning and memory function in mice with SAE by promoting autophagy in the hippocampus; and (c) SESN2 regulated the AMPK/mTOR signalling pathway, promoting ULK1‐dependent autophagy in neurons.

Previously, deregulated SESN2 expression was demonstrated in various disease processes, including UVB stress. SESN2 was detected in human skin fibroblasts and keratinocytes after acute exposure to high and low doses of low‐linear energy transfer (LET) ionizing radiation.^24^ SESN2 was also significantly up‐regulated in skin tissue and sepsis.^25^ These studies showed that extended lipopolysaccharides (LPS) increased SESN2 expression *via* NOS2 (nitric oxide synthase 2, inducible)‐mediated NO (nitric oxide) in macrophages.^26^ The present study demonstrated that SESN2 was initially detected in the hippocampi of mice with CLP‐induced SAE, and further staining indicated its up‐regulation in hippocampal neurons. As previous reports showed that P53,^14^ lysine‐specific demethylase LSD1,^27^ and activating transcription factor 4 (ATF4) ^28^ are SESN2 transcriptional regulators, we speculated that ATF4, which was elevated by brain‐derived neurotrophic factors, may be the crucial inductor of SESN2 up‐regulation in hippocampal neurons during SAE. Therefore, further studies are warranted to validate this hypothesis.

The adenovirus‐based SESN2 overexpression system in the galactosamine (Gal)/LPS‐induced liver injury model decreases ALT, AST and hepatocyte degeneration *via* inhibition of the TLR‐induced pro‐inflammatory signalling pathway in macrophages.^29^ Additionally, SESN2 knockdown aggravates atherosclerotic processes by increasing pro‐inflammatory reactions and ER stress in the endothelium.^30^ Moreover, SESN2 also controls the ROS‐dependent neuropathic pain signalling pathway following peripheral nerve injury.^31^ Furthermore, it critically mediates hepatocellular adaptation to ER stress and functions as a crucial endogenous attenuator of non‐alcoholic fatty liver disease (NAFLD) progression.^32^ In the current study, AAV2 (an efficient delivery system for brain disease gene therapy ^33^) was utilized to regulate SESN2 expression in mouse hippocampi. SESN2 ectopic expression attenuated brain damage and loss of learning and memory function in mice with CLP‐induced SAE. In addition, decreased pro‐inflammatory cytokine expression and inflammatory cell infiltration were observed in hippocampi of AAV2‐SESN2‐injected mice with SAE, consistent with the anti‐inflammatory role of SESN2 in sepsis.^26^ Further studies are required to elucidate the direct cause of tissue damage regulated by SESN2 expression.

Interestingly, autophagy modulation appears to protect against multiple organ injuries in murine sepsis models,^7^, ^8^ and SESN2 promotes autophagy development in macrophages,^26^ cancer cells^18^ and myotubes.^34^ In the present investigation, we demonstrated that SESN2 induced ULK1‐dependent autophagy in the hippocampal neurons of mice with SAE, thus playing an essential role during SESN2‐mediated SAE attenuation. Although several kinase‐related pathways, including the AMPK/mTOR,^19^ p38‐c‐Jun^29^ and Akt^35^ signalling pathways are downstream targets of SESN2, we demonstrated that the Akt pathway regulated activation of the AMPK/mTOR signalling pathway, a necessary step during SESN2‐induced autophagosome formation and SAE protection in mice.

In summary, our findings indicated an up‐regulation of SESN2 in the hippocampal neurons of mice with SAE and attenuation in SAE development through the promotion of ULK1‐dependent neuron autophagy following SESN2 ectopic expression. Therefore, we propose SESN2 as a novel pharmacological intervention strategy for SAE treatment.

## CONFLICT OF INTEREST

The authors declare that they have no potential conflicts of interest.

## AUTHOR CONTRIBUTIONS

Deyuan and Lili conducted all the experiments. Lina and Lili participated in the design of the study and helped to draft the manuscript. Guoyan conducted the statistical analysis. Jinlin and Jinhui participated in the physiological examination. Deyuan and Lili designed the project and finalized the manuscript. All authors read and approved the final manuscript.

## ETHICS APPROVAL AND CONSENT TO PARTICIPATE

All experimental procedures were approved by the Institutional Animal Care and Use Committees of Sichuan University.

## CONSENT FOR PUBLICATION

Consent for publication is not applicable in this study; no individual person's data were used.
